# *Toxoplasma gondii* Decreases the Reproductive Fitness in Mice

**DOI:** 10.1371/journal.pone.0096770

**Published:** 2014-06-18

**Authors:** Katerina Dvorakova-Hortova, Adela Sidlova, Lukas Ded, Denisa Hladovcova, Markus Vieweg, Wolfgang Weidner, Klaus Steger, Pavel Stopka, Agnieszka Paradowska-Dogan

**Affiliations:** 1 Biocev Group, Department of Zoology, Faculty of Science, Charles University in Prague, Prague, Czech Republic; 2 Laboratory of Reproductive Biology, Institute of Biotechnology, Academy of Science, Prague, Czech Republic; 3 Department of Urology, Pediatric Urology and Andrology, Justus Liebig University of Giessen, Giessen, Germany; Obihiro University of Agriculture and Veterinary Medicine, Japan

## Abstract

*Toxoplasma gondii* is a common protozoan parasite that infects warm-blooded animals throughout the world, including mice and humans. During infection, both, the parasite and the host, utilize various mechanisms to maximize their own reproductive success. Mice and humans are both the intermediate hosts for *Toxoplasma gondii*, which forms specialized vacuoles containing reproductive cysts in the formers’ tissue. As half of the human population is infected, developing a disease called toxoplasmosis, along with an ever-growing number of couples suffering with idiopathic infertility, it is therefore surprising that there is a lack of research on how *Toxoplasma gondii* can alter reproductive parameters. In this study, a detailed histometric screening of the testicular function along with the levels of the pituitary luteinizing hormone (LH) were analysed in infected mice. Data on relative testis and epididymis weight, and sperm count were also collected. Based on the results obtained, the level of LH in the urine of *Toxoplasma gondii* infected mice was lower compared to the control. In direct correlation with the hormone level, testicular function and sperm production was also significantly lower in *Toxoplasma gondii* positive group using sperm count and histometric analysis as a marker. Not only were the number of leptotene primary spermatocytes and spermatids lowered, but the number of Sertoli cells and the tubule diameter were elevated. In parallel, a pilot epigenetic study on global testicular methylation, and specific methylation of Crem, Creb1 and Hspa1genes essential for successfully ongoing spermatogenesis was performed. Global methylation was elevated in *Toxoplasma* infected mice, and differences in the DNA methylation of selected genes were detected between the *Toxoplasma* positive and control group. These findings demonstrate a direct relation between *Toxoplasma gondii* infection and the decrease of male reproductive fitness in mice, which may contribute to an increase of idiopathic infertility in humans.

## Introduction

Toxoplasmosis, caused by the protozoan parasite *Toxoplasma gondii* (*T. gondii*), is one of the most common parasitic infections of man and other warm-blooded animals. Nearly one-third of humanity has been exposed to this parasite [1]. In the United States and the United Kingdom, it is estimated that 16–40% of the population are infected, whereas in Central and South America and continental Europe, estimates of infection range from 50 to 80% [2]. There are different types of strains of *T. gondii.* Most *Toxoplasma* strains isolated in North America and Europe belong to three clonal lines referred to as: Type I, II and III [3], [4]. Type I strains are hypervirulent and infection causes death in immunocompetent mice. Type II and III avirulent strains cause nonlethal infections, characterized by the development of chronic latent infections of the CNS and skeletal muscles. In most human adults *T. gondii* does not cause serious illness, however, getting infected during pregnancy may cause devastating consequences for a developing fetus [5]. Furthermore connections were made between *Toxoplasma* infection and the sperm quality in humans [6]. It was later documented [7] that there was a decreased fertility in rats after being infected with *T. gondii* along with a lower weight of epididymis, decreased sperm motility and concentration, and an increased number of abnormal sperm morphology. Additionally, the correlation between toxoplasmosis and increased sperm apoptosis, especially of diploid spermatocytes, was described [8].

The mechanism by which *T. gondii* alters reproductive parameters is not known yet. The one suggested pathway is by hormonal regulation through the level of gonadotropins (LH and FSH), which regulate the entire process of spermatogenesis. Long-term stress that is triggered by an ongoing infection, inhibits the activity of the hypothalamus-hypophysis axis by releasing stress hormones that lead to a decrease of LH release, therefore consequently to a decrease of spermatogenesis [9]. *T. gondii* may, through peripherally circulating cytokines, increase a release of corticotropin-releasing factor (CRF, a neuropeptide regulating stress response) by hypothalamic neurons and in response inhibit the release of gonadotropin-releasing hormone (GnRH) from hypothalamus [7]. This cascade directly leads to pituitary gonadotropin insufficiency. However, this suggested mechanism has not been tested further yet and a connection between toxoplasmosis and a modified level of gonadotropins has not been shown.

In the first stage of this work, we tested the urine level of LH over a 30-day *Toxoplasma* infection period in mice, and through further detailed morphometric analysis of testicular tissue, we examined the quality of spermatogenesis after this acute state of the infection.

The second aspect, how an infection can modify the host reproductive parameters, is through the epigenetic route, which alters the methylation of certain specific genes regulating the spermatogenesis and therefore the gene expression. Co-evolution of pathogens and eukaryotic cells enables the pathogen to utilize host cells to survive, replicate and escape the immune system [10]. Pathogens can even modify the host cell immunological reaction by manipulating the epigenetic processes, which can lead to the development of a chronic form of the infection [11]. Beside viruses and bacteria, micro-parasites, such as protozoa, as well as macro-parasites and environmental factors can trigger pathological changes in the epigenome of infected host cells. They can hyper or hypo-methylate specific key gene promoters including those responsible for ongoing spermatogenesis [12], [13]. However, certain patho-epigenetic changes are reversible or avoidable if well understood, which is of major therapeutic importance [14]. Abnormal DNA methylation in male germ cells triggers aberration of testicular histology and altered spermatogenesis in mice and humans [12], [15]. Such genes, of which the expression is regulated by both gonadotropins and promoter methylation, are Crem and Creb1 genes coding the cAMP response element (CRE), which is a key regulator of spermatogenesis [16]. CREM (cAMP response element modulator) and CREB (cAMP response element binding protein) are cyclically expressed at high levels during spermatogenesis. CREM and CREB represent transcriptional factors involved in the cAMP signalling pathway, which regulate the expression of several spermatogenesis specific genes. These act as important hormone-responsive regulators of mammalian cell growth, differentiation and survival during spermatogenesis. Both CREB and CREM specifically bind to the CRE element (cAMP-response element) in the promoter of important target genes crucial for germ cell development, and they are expressed at different times of spermatogenesis. They undergo programmed sequential switches from being an activator to being a repressor of isoforms by mechanisms of alternative exon splicing and promoter usage, and are autoregulated by cAMP signaling in opposing directions [17].

An abnormal Crem methylation in men with oligozoospermia along with a decreased sperm motility and quality was reported [18]. A similar outcome is likely to be observed for the epigenetically modified function of the Hspa1 gene, which encodes the testes specific heat-shock cognate protein Hsc70T, a member of the heat-shock Hsp70 protein family. The Hsc70T protein is expressed in germ cells during post-meiotic differentiation and its deficiency leads to lower sperm motility [19]. Also, Creb314-deficient mice (Creb314 belongs to the CREB family of transcription factors) displayed an increased apoptosis of meiotic and postmeiotic male germ cells, however, the fertility was not compromised [20]. These phenotypical defects are in correlation with defects described after *T. gondii* infection and can serve as a link between the infection and its mechanism [7].

In the second part of this work, we focused on testing global testicular methylation in the *T. gondii* positive and control group, and then we analysed the specific methylation of three selected genes Crem, Creb1 and Hspa1, essential for successful spermatogenesis and at the same time being responsive to epigenetic regulation through DNA methylation.

Bringing both histometrical and epigenetical studies together, *T. gondii* modifies the release of gonadotropin hormones and may also alter the testicular epigenome. Consequently the parasite may amend the spermatogenesis and decrease reproductive parameters and male reproductive fitness. Therefore, the mechanism through which *T. gondii* targets and modifies its host homeostasis seems to be complex and it deserves to be further investigated.

## Materials and Methods

### Ethics Statement

All animal procedures were carried out in strict accordance with the law of the Czech Republic, paragraph 17 no. 246/1992, and Animal Scientific Procedures paragraph 11, no. 207/2004.The local ethics committee of the Faculty of Science of Charles University in Prague specifically approved this study in accordance with accreditation no. 24773/2008-10001.

### Animals

A total of 18 male C57Bl/6 mice were purchased from Anlab (Czech Republic) at the age of 60 days and used as subjects for this study. Mice were housed in The National Institute of Public Health, Prague, Czech Republic. There were 3 males in each cage, both prior and during the whole experiment, with a controlled 12-hour light-dark cycle and a stable room temperature (19–21°C). Food and water were supplied ad libitum. The experiment was started after a 10-day adjustment period, when mice were 70 days of age. 9 males were randomly selected as a control *Toxoplasma* negative (Toxo^−^) group. The same number of males was infected according to the infection procedure in the paragraph below and these established the experimental *Toxoplasma* positive (Toxo^+^) group. Urine samples were taken on the day before the infection procedure started (Day 69), and on the last day of the experiment (Day 100), stored at −20C and used for LH level analysis. Mice were killed by cervical dislocation 30 days after being infected at the age of 100 days. The body weight, as well as the weight of the right and left testis and epididymis, was taken.

The testes were frozen at −80°C and further used for detailed histometric examination and DNA methylation analysis. Blood serum from each mouse was prepared for the assessment of infection positivity (see infection procedure). There was an accidental mortality in each group, so at the end, 8 males in each group were analysed.

### Infection Procedure

The grouped mice were left to acclimatize for a period of 7 days prior to the experiment. At the time of infection the males were 70 days old and 20 g±3 g of weight. From the total of 18 male mice, 9 individuals served as the control group, and the other 9 were infected with the human origin *T. gondii* avirulent type II strain HIF, cyst-forming isolate [21]. Experimental individuals were infected orally with a homogenized mouse brain solution containing 15 tissue cysts in a buffer saline. The same amount of non-infected solution was administered to the controls. The presence of the infection was confirmed by CFR (complement fixing method [22]) from sera at the end of the experiment. Sera were tested at the National Reference Laboratory for Toxoplasmosis, National Institute of Public Health, Prague, Czech Republic. All experimental mice were successfully infected and had CFT titre higher than 1∶260.

### Epididymal Sperm Concentration

Spermatozoa from the fifth region of the left and right cauda epididymis were released into 100 µl of Tris buffer saline (TBS) (pH 7.34) each for 5 minutes at 37°C. The concentration of spermatozoa in 1 ml was assessed in a haemocytometer chamber according to a standard protocol under 100x magnification.

### Histometric Analysis

Testes were frozen after separation in liquid nitrogen and stored at −80°C. One testis from each individual was used for histometric analysis. Frozen testes were inserted into 30% sucrose liquid solution for 24 hours, further embedded in a tissue tack and sectioned by cryotome at a thickness of 7 µm. All sections were fixed by acetone, stained with hematoxylin-eosin, covered with Canadian balsam and examined with an Olympus BX51 light microscope.

Each testis analysis included measurements of the minor diameter of 50 seminiferous tubules (TD), tallies of the number of Sertoli cells (SC), leptotene primary spermatocytes (LS) and spherical spermatids (S) within 20 seminiferous tubular cross sections and tallies of the number of primary leptotene primary spermatocytes per 250 Sertoli cells (index250).

### LH Detection

LH levels were measured in urine samples collected on Day 0 (the day prior the infection procedure) and on Day 30 (the final day of the experiment). A ST AIA-PACK LH II kit, (cat. no 0025296, Medesa, CZ), was used for the quantitative determination of the luteinizing hormone (LH) in urine. The kit was a two-site immune-enzymometric assay, which has been performed entirely in AIA-PACK test tubes. LH present in the test sample was bound to a monoclonal antibody immobilised on magnetic beads and an enzyme-labelled monoclonal antibody in the AIA-PACK test. The magnetic beads were washed to remove any non-bound enzyme-labelled monoclonal antibodies and then incubated with the fluorogenic substrate 4-methylumbelliferylphosphate (4 MUP). The amount of enzyme-labelled monoclonal antibody that binds to beads was in direct proportion to the LH concentration in the test sample. A standard curve has been constructed, and unknown sample concentrations were calculated using this curve. The Tosoh AIA System Analyser (Tosoh Bioscience, USA) was used to read the rate of the fluorescence produced by the reaction, and to automatically convert the rate to a LH concentration in mIU/ml (protocol ST AIA–PACK LH II).

### DNA Isolation and Sodium Bisulfite Conversion

DNA was isolated from testicular tissue using a GenElute TM Mammalian Genomic DNA Miniprep kit, (cat. no G1N350-1KT, Sigma-Aldrich, CZ). The tissue is lysed in a chaotropic salt containing solution to insure the thorough denaturation of macromolecules. The addition of ethanol causes DNA to bind when the lysate is spun through a silica membrane in a microcentrifuge tube. After washing to remove contaminants, DNA is eluted in 200 mL of Tris-EDTA solution (protocol GenElute™ Mammalian Genomic DNA Miniprep kit).

A DNA Modification Kit (cat. no MOD50-1KT, Sigma Aldrich, CZ) was used for the bisulphite conversion of DNA. DNA denaturation and bisulfite modification are carried out simultaneously. In the modification process, bisulfite reacts specifically with single-stranded DNA to deaminate the cytosine, creating a uracil residue. The unique DNA protection reagents in the modification buffer prevent the chemical and thermophilic degradation of DNA in the bisulfite treatment. The Capture Solution enables DNA to tightly bind to the column filter. This enables the effective removal of residual sodium bisulfite and salts (protocol DNA Modification kit).

### Analysis of Global DNA Methylation Quantification using ELISA

Analysis of global DNA methylation was performed using a Methylated DNA quantification kit, (cat. no MDQ1-48RXN, Sigma Aldrich, CZ), with a format similar to sandwich ELISA assay. The methylated DNA is detected by using the capture and detection antibody, and the results are quantified calorimetrically. The detection antibody binds to the enzyme cleaving colourless substrate, which is present in the developing solution, to the coloured product. The amount of methylated DNA present in the sample is proportional to the absorbance measured.

### Quantitative Analysis of CpG Methylation by Pyrosequencing

To quantify the methylation of a single CpGs, 20 ng of bisulphate treated DNA was amplified by PCR with specific primers. These primers were part of ready-to-use assays provided from Qiagen. We used the following assays: Mm_Crem_02_PM (PM00306943), Mm_Creb1_01_PM (PM00205142), Mm_Hspa1a_01_PM (PM00291004). The PCR reaction contained 12.5 µl PyroMark PCR Master Mix (Qiagen), 2.5 µl CoralLoad (Qiagen) and 2.5 µl of 0.2 µM primer mix together with DNA diluted with ddH2O at a final volume of 25 µl. The following conditions were used for PCR: 95°C for 15 minutes then cycled 45 times at 95°C for 30 seconds, 56°C for 30 seconds and 72°C for 30 seconds and a final extension at 72°C for 10 minutes. The PCR products were pyrosequenced in a Qiagen PyroMark Q24. For that we used the standard protocol and the required chemicals supplied by Qiagen. The sequencing primers were part of the previously described assays. The received data were analysed by Qiagen Q24 Software.

### Statistical Analysis

Experimental data were analysed using STATISTICA 6.0 and GraphPad Prism 5.04. The differences in the number of individual cell types and the tubular diameter between the Toxo^+^ and Toxo^−^ group were tested by Kruskal–Wallis one-way analysis of variance (KW-ANOVA). The differences in LH level, body and organ weights between Toxo^+^ and Toxo^−^, were analysed by t-test. The differences in the global DNA methylation between Toxo^+^ and Toxo^−^ were analysed by the repeated measures ANOVA test. The differences in the specific methylation of Crem, Creb1 and Hspa1 were analysed by one-way ANOVA, and the Newman-Keuls Multiple Comparison test was used as post-hoc analysis. The p value equal or lower than 0.05 was considered to be significant, *p value≤0.05 (**p≤0.01 and ***p≤0.001).

## Results

### Body and Reproductive Organ Weights

**Figure 1:**
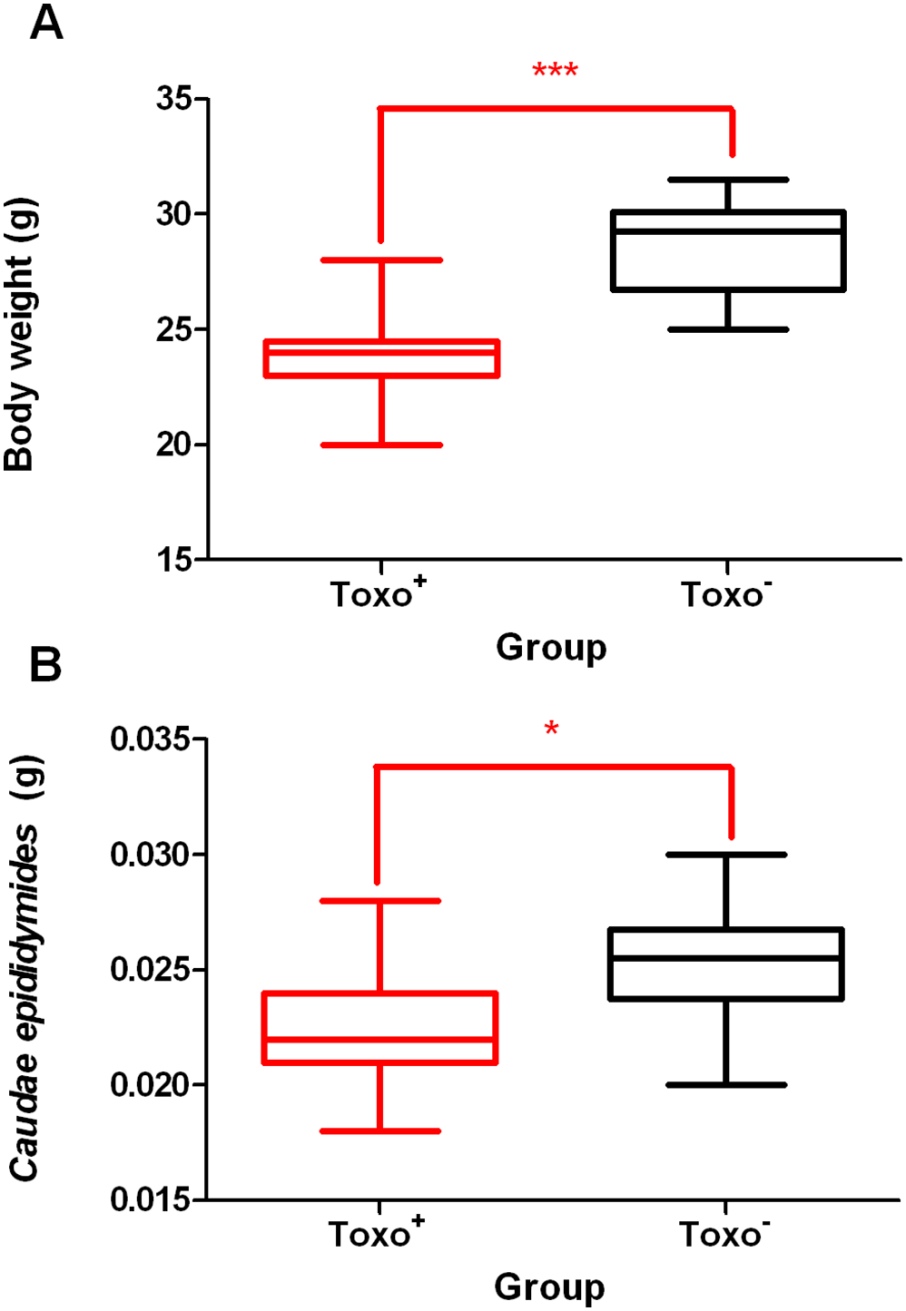
A The body weight comparison between Toxo^+^ and Toxo^−^ group. The middle line indicates the arithmetic mean, the box extends from the 25th to 75th percentiles and the whiskers indicate the minimum and maximum of the measurement. (***p≤0.001). **B** The caudae epididymidae weight comparison between Toxo^+^ and Toxo^−^ group. The middle line indicates the arithmetic mean, the box extends from the 25th to 75th percentiles and the whiskers indicate the minimum and maximum of the measurement. (*p value≤0.05).

There were significant differences between Toxo^+^ and Toxo^−^ in body weight (***p≤0.001) (23.95±1.93 vs. 28.70±2.06) (Figure 1A) and the *cauda epididymis* weight (0.0227±0.0028 vs. 0.0254±0.0028) (*p≤0.05) (Figure 1B.) There were no statistically significant differences found in the weight of testes (0.1673±0.0199 vs. 0.1671±0.0376) (p≥0.05) between the Toxo^+^ and Toxo^−^ group. However, the same trend in testis weight loss was seen.

### Epididymal Sperm Concentration

**Figure 2:**
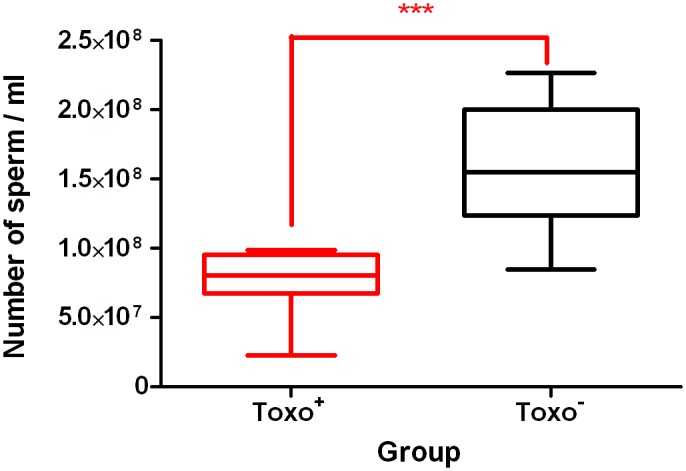
The sperm concentration comparison between Toxo^+^ and Toxo^−^ group. The middle line indicates the arithmetic mean, the box extends from the 25th to 75th percentiles and the whiskers indicate the minimum and maximum of the measurement. (***p≤0.001).

The epididymal sperm concentration was significantly lower in the Toxo^+^ group compared to the Toxo^−^ group (0.7.5×10^89^±0.2.4×10^89^ vs. 1.6×10^810^±0.4.4×10^89^) (***p≤0.001) (Figure 2). The data obtained positively correlate with the decreased weight of the caudae epididymides in the Toxo^+^ group, which was significant (Figure 1B).

### Histometric Analysis of Testicular Function

**Figure 3:**
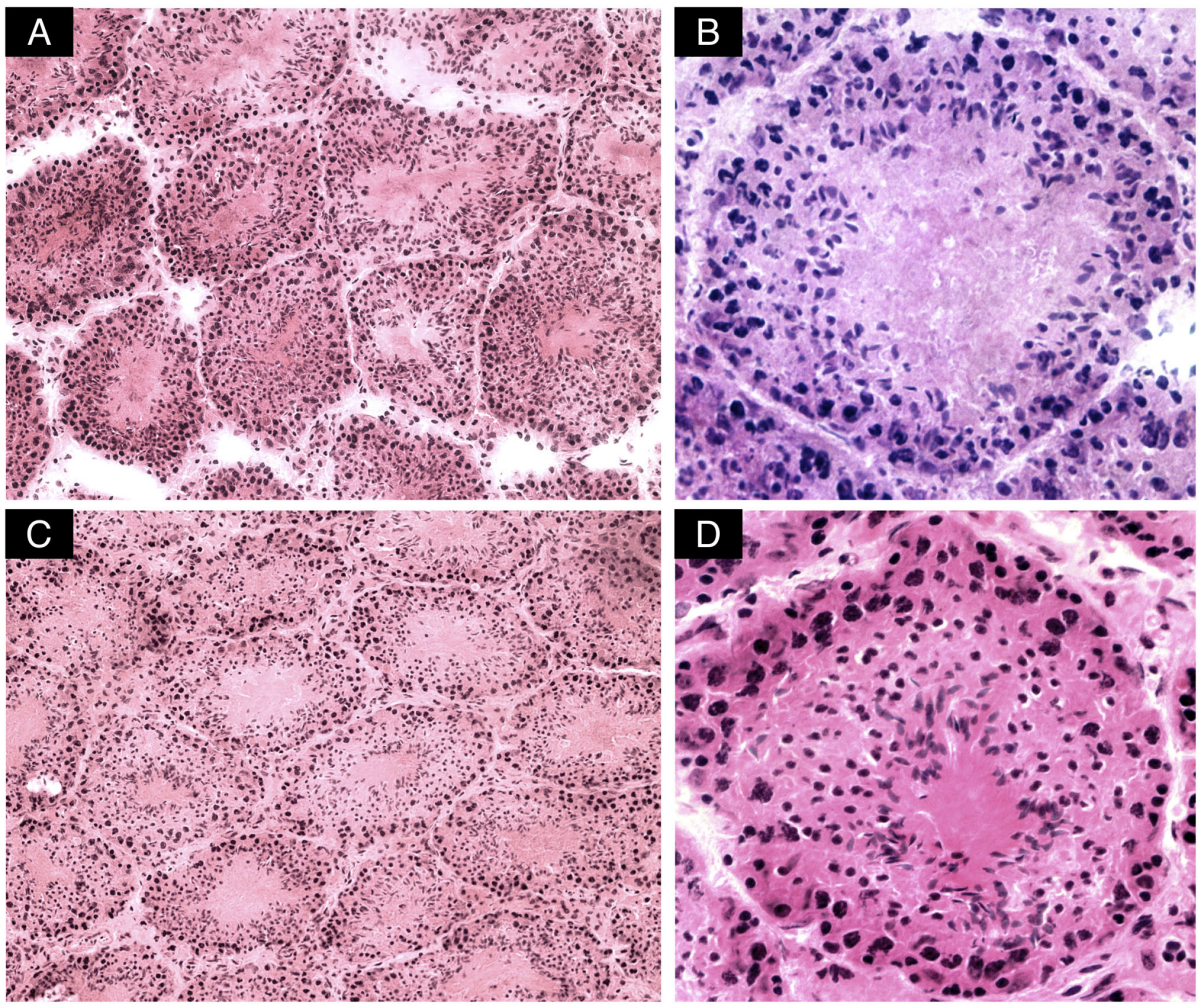
Cross sections of *tubuli seminiferi* from mice testes infected with *Toxoplasma gondii.* No dramatic effect on the histological architecture could be observed in the testis of infected mice compared to controls. Magnifications 20x (A) and 40x (B) and control samples 20x (C) and 40x (D).

**Figure 4:**
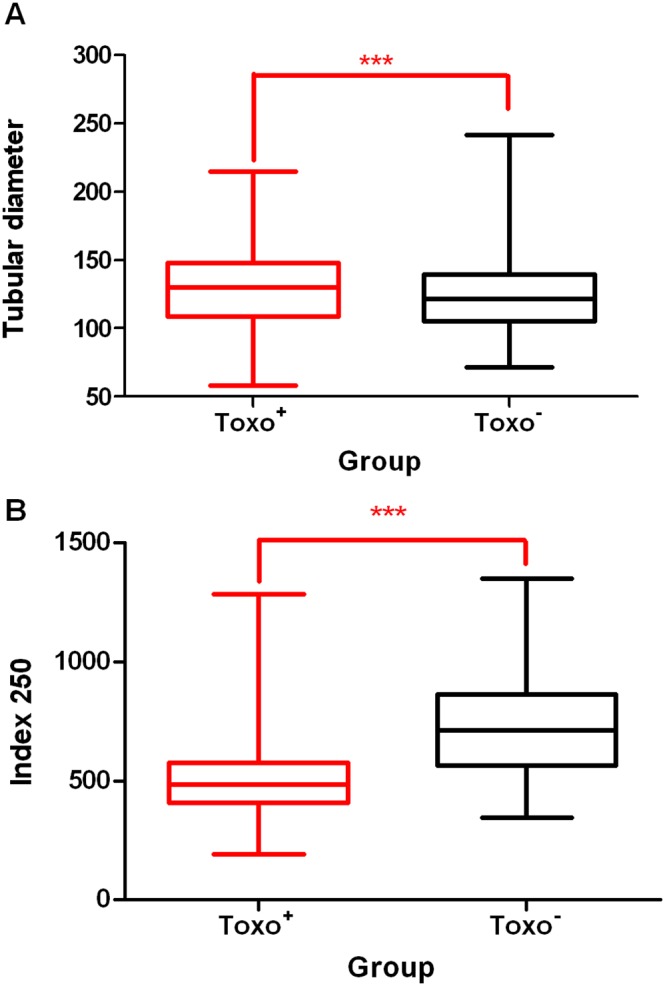
A The tubular diameter comparison between Toxo^+^ and Toxo^−^ group. The middle line indicates the arithmetic mean, the box extends from the 25th to 75th percentilesand the whiskers indicate the minimum and maximum of the measurement. (***p≤0.001). **B** The differences in the Index 250 between Toxo^+^ and Toxo^−^ group. The middle line indicates the arithmetic mean, the box extends from the 25th to 75th percentiles and the whiskers indicate the minimum and maximum of the measurement. (***p≤0.001).

**Figure 5:**
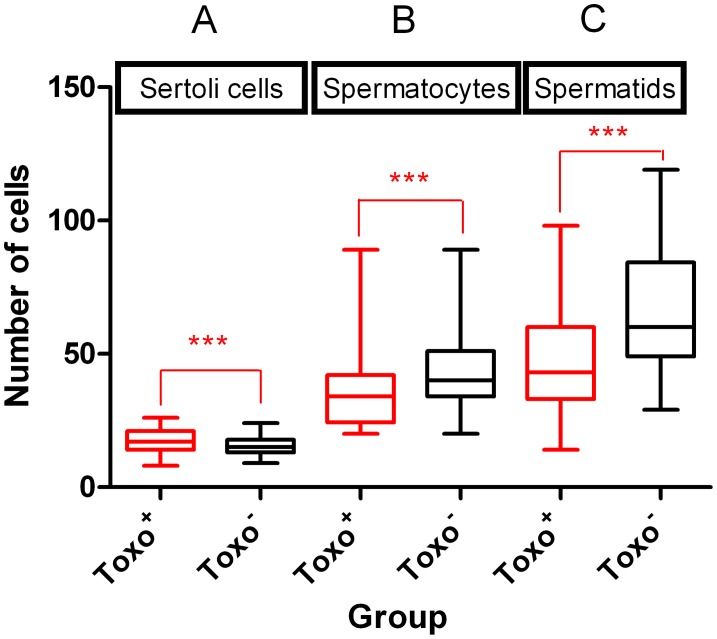
Histometric analysis of testicular cells. The number of Sertoli cells (A), Leptotene primary Spermatocytes (B), Spermatids (C) in Toxo^+^ and Toxo^−^ group. The middle line indicates the arithmetic mean, the box extends from the 25th to 75th percentiles and the whiskers indicate the minimum and maximum of the measurement. (***p≤0.001).

Representative cross-sections of testicular tubules from Toxo^+^ and Toxo^−^ mice are demonstrated in Figure 3. The results of histometric analysis are presented in Figure 4A, 4B, 5 and Table S1. The average number of Sertoli cells (SC) (Figure 5), leptotene primary spermatocytes (LS), spermatids (S), and the minor diameter of the seminiferous tubule (TD) (Figure 4A) in Toxo^+^ and Toxo^−^ were statistically analysed. There were statistically significant differences (***p<0.001) between both groups in all parameters. For the Toxo^+^ group, there was a statistically higher amount of Sertoli cells (17.61±4.03 vs. 15.15±3.22) and a larger tubule diameter (129.68±32.02 vs. 125.78±30.26) and at the same time a lower number of leptotene primary spermatocytes and spermatids compared to the Toxo^−^ group.

The ratio of leptotene primary spermatocytes per 250 Sertoli cells was established for each experimental animal (Index250) (Figure 4B). The ratio was significantly reduced (528.47±214.44 vs. 675.79±175.63) (***p<0,001) in the Toxo^+^ group compared to the Toxo^−^ group.

**Figure 6:**
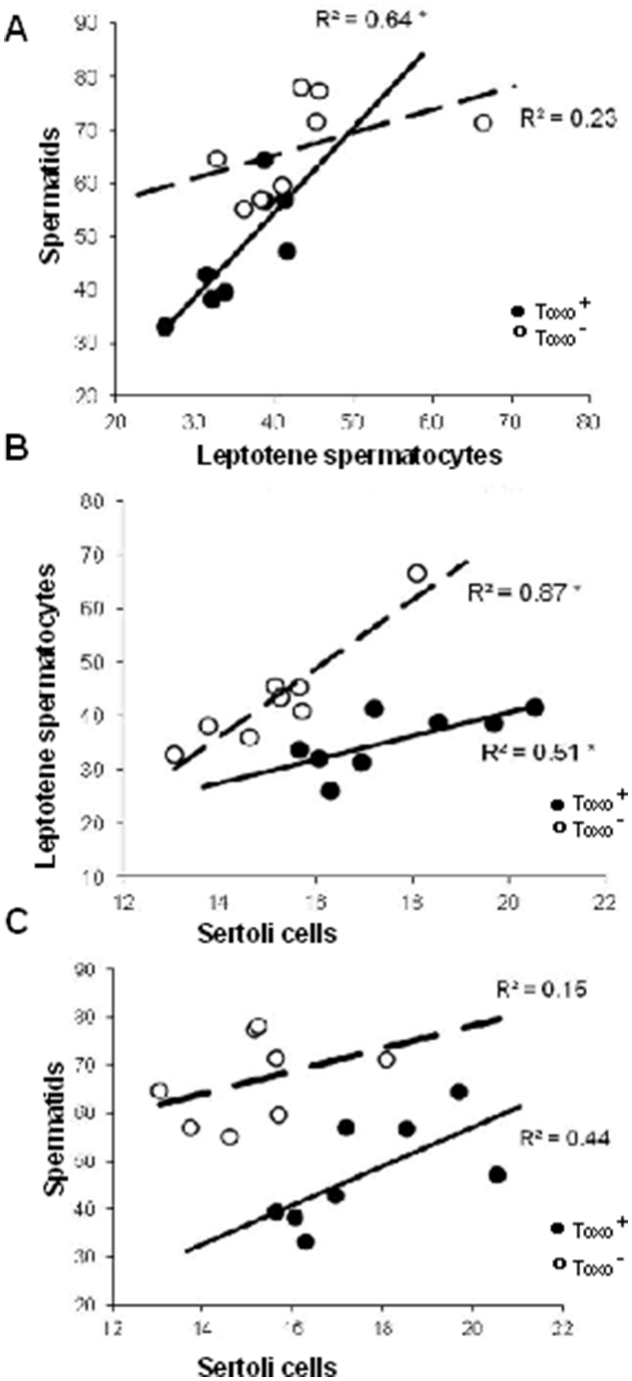
Graphs showing a level of correlation between individual parameters within Toxo^+^ and Toxo^−^ group. Sertoli cells/Leptotene primary spermatocytes (A), Sertoli cells/spermatids (B) Leptotene primary Spermatocytes/Spermatides (C) The statistically significant coefficient of determination R^2^ is indicated by an asterix*.

Linear regression analysis was applied to analyse the level of correlation between individual obtained measurements of selected histometric parameters within Toxo^+^ and Toxo^−^ group (Figure 6 A, B, C). The statistically significant coefficient of determination R^2^ is in regression models shown by aster. The correlation between parameter LS/S (Figure 6A) and SC/LS (Figure 6B) was significant within both the Toxo^+^ and Toxo^−^ group, with a strong relationship between two evaluated continuous variables in Toxo^+^ and Toxo^−^ group for SC/LS parameters (Figure 6B) and in Toxo^+^ group for LS/S parameters (Figure 6A). On the other hand, the correlation between SC/S (Figure 6C) did not show a statistical significance in the data in either group suggesting that the number of Sertoli cells may not reflect the final number of maturing spermatids in the used histometric analysis.

Using computed modelling, there were visible differences in the number of spermatids dependant on the number of Sertoli cells and spermatocytes between the Toxo^+^ and Toxo^−^ group (Figure S1). The model distributions show that in the Toxo^−^ group, the number of spermatids is increasing together with the number of spermatocytes. On the other hand, in the Toxo^+^ group, there is Gaussian-like distribution of the number of spermatids is dependent on the number of spermatocytes.

### LH Detection

**Figure 7:**
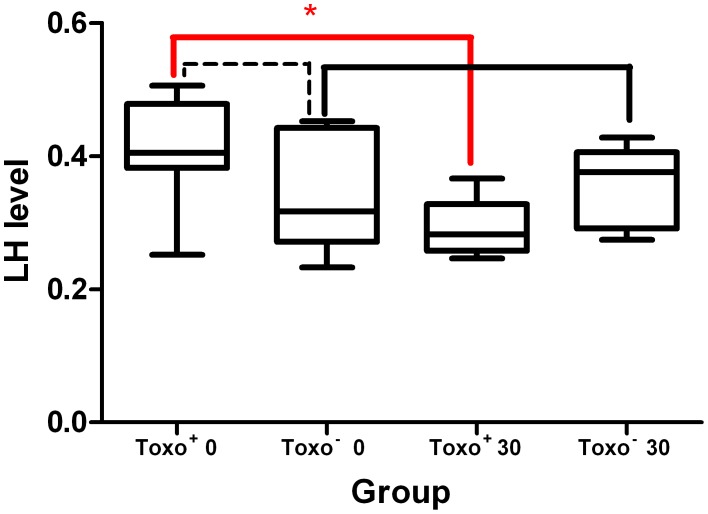
The LH level comparison in urine of Toxo^+^ and Toxo^−^ at Day 0 (the day prior) and Day 30 (at the end) of the experiment. The middle line indicates the arithmetic mean, the box extends from the 25th to 75th percentiles and the whiskers indicate the minimum and maximum of the measurement. The broken line indicates no significant difference between the Toxo^+^ and Toxo^−^ group at the beginning of the experiment. (*p value≤0.05).

The data summarizing levels of LH (random units) in mouse urine before and after the infection in Toxo^+^ and control Toxo^−^ are shown in Figure 7. The results show that there was a statistically significant difference in the LH levels within the Toxo^+^ group between the phase before and after the infection, where the LH level had decreased (0.407±0.082 vs. 0.291±0.042) (*p≤0.05) in the group after the 30-day infection period. There were no significant differences within the Toxo^−^ group (0.339±0.084 vs. 0.360±0.057) (p≥0.05) in the same parameter.

### Testicular Global DNA Methylation

**Figure 8:**
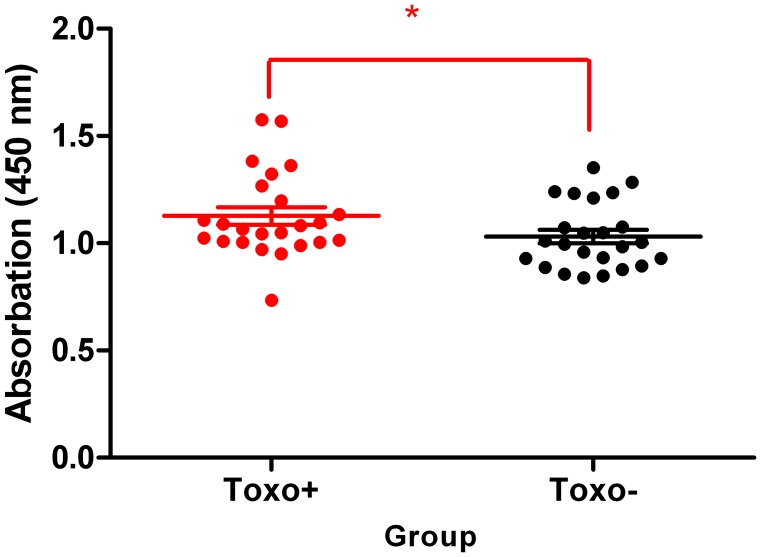
Effect of *Toxoplasma gondii* on testicular global DNA methylation. The middle line indicates the arithmetic mean, the whiskers indicate standard deviation and points indicate individual measurements. (*p≤0.05).

Global DNA methylation quantification served as the first indicator whether or not *T. gondii* may trigger global regulatory changes in genome methylation. Using DNA isolated specifically from testicular tissue, the global DNA modification of testicular epigenom was targeted. According to the obtained measurements, statistically significant differences in the global DNA methylation were detected between the Toxo^+^ and Toxo^−^ group (1.35±0.16 vs. 1.19±0.13) (**p≤0.01). Also, the testicular global methylation was higher in the Toxo^+^ group compared to the control (Figure 8).

### Quantitative Analysis of CpG Methylation of Selected Genes Regulating Spermatogenesis

**Figure 9:**
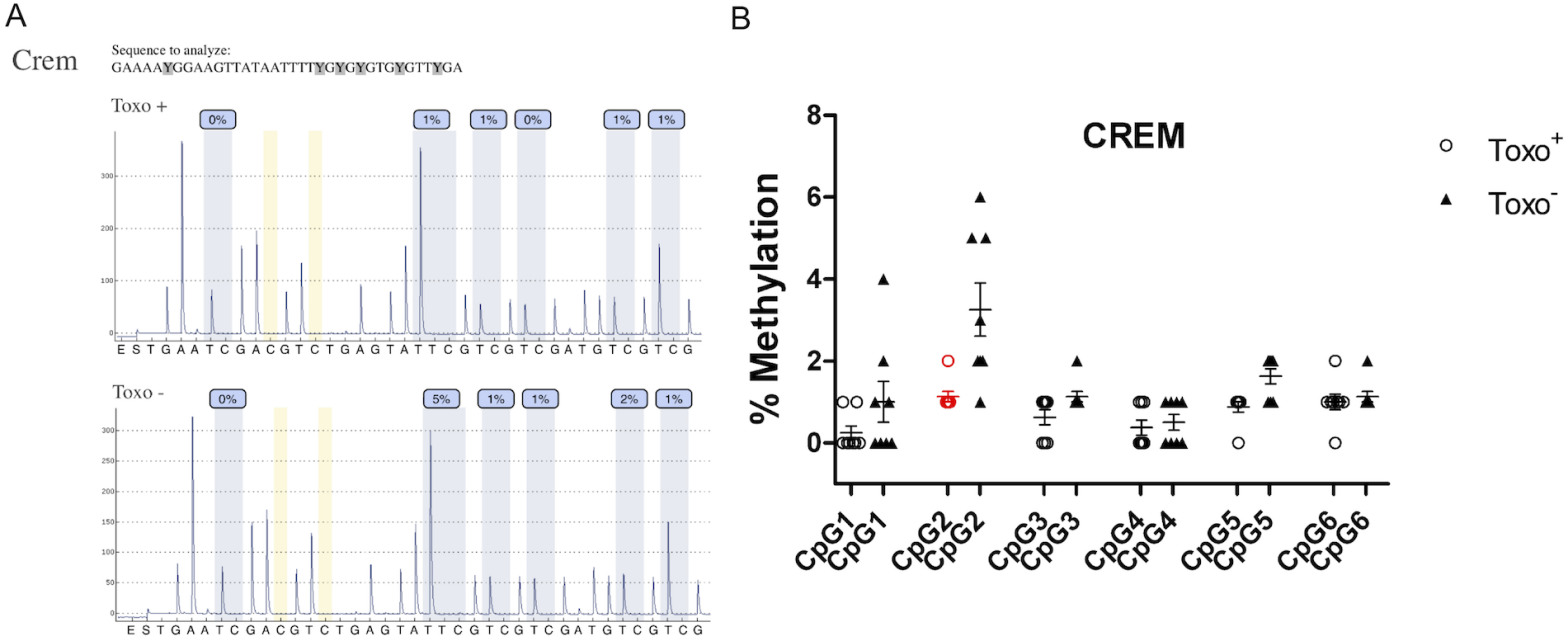
Quantitative analysis of CpG methylation of the Crem gene promoter in Toxo^+^ and Toxo^−^ group. A. An exemplary pyrogram showing the methylation level in each CpG between the representative Toxo^+^ and Toxo^−^ samples. B. Summary of methylation in Toxo^+^ and Toxo^−^ groups. The middle line indicates the arithmetic mean, the whiskers indicate the standard deviations and the points indicate individual measurements (% of methylation of the appropriate CpG position). The red color indicates the significant difference (*p value≤0.05).

**Figure 10:**
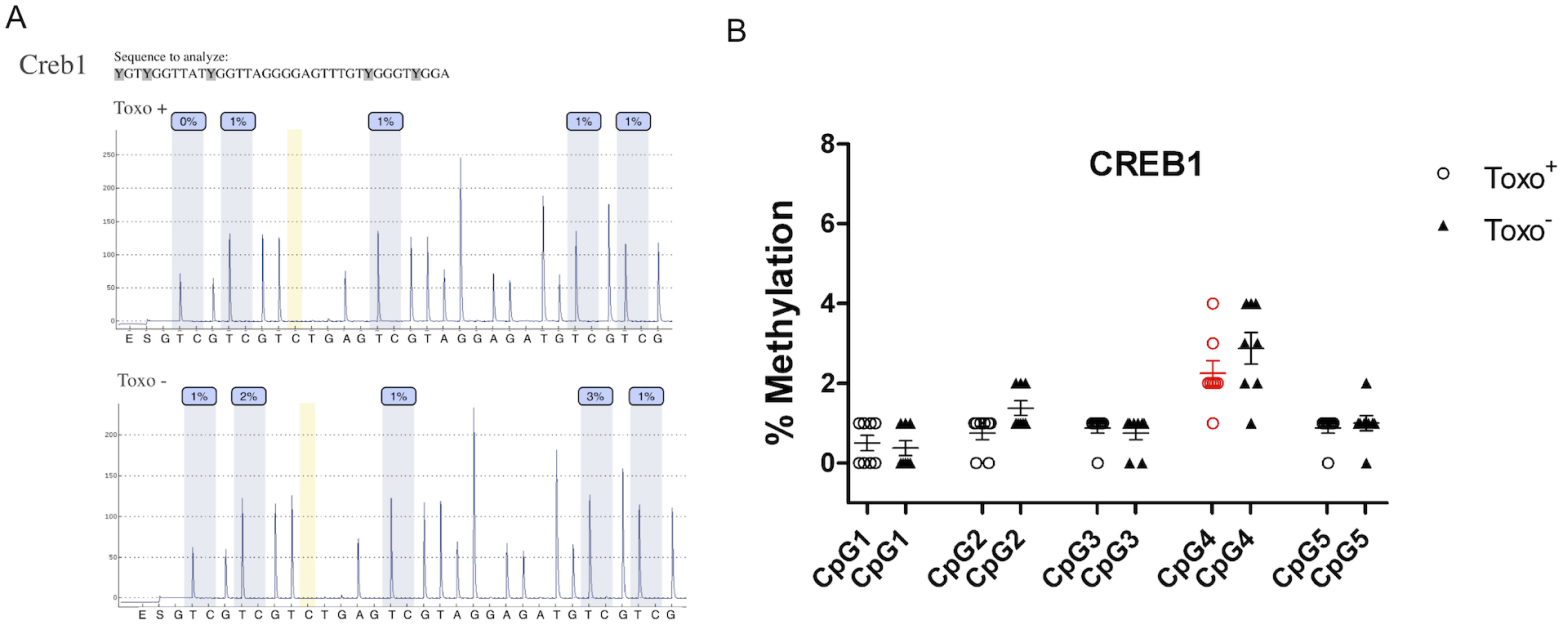
Quantitative analysis of CpG methylation of the *Creb1* gene promoter in Toxo^+^ and Toxo^−^ group. A. An exemplary pyrogram showing the methylation level in each analysed CpG between the representative Toxo^+^ and Toxo^−^ samples. B. Summary of methylation in Toxo^+^ and Toxo^−^ groups. The middle line indicates the arithmetic mean, the whiskers indicate the standard deviations and the points indicate individual measurements (% of methylation of the appropriate CpG position). The red color indicates the significant difference (*p value≤0.05).

**Figure 11:**
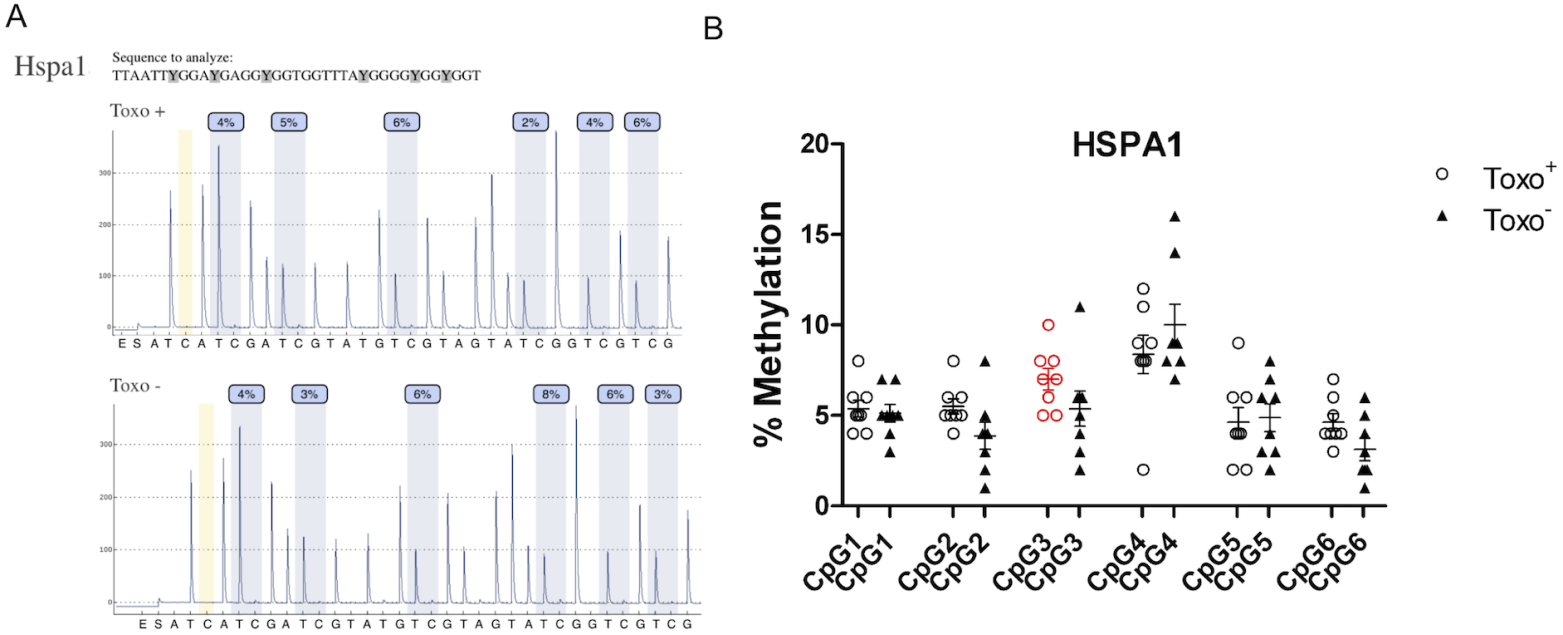
Quantitative analysis of CpG methylation of Hspa1 gene promoter in Toxo^+^ and Toxo^−^ group. A. An exemplary pyrogram showing the methylation level in each analysed CpG between the representative Toxo^+^ and Toxo^−^ samples. B. Summary of methylation in Toxo^+^ and Toxo^−^ groups. The middle line indicates the arithmetic mean, the whiskers indicate the standard deviations and the points indicate individual measurements (% of methylation of the appropriate CpG position). The red color indicates the significant difference (*p value≤0.05).

The quantitative analysis of specific DNA methylation was detected for *Crem* (Figure 9), *Creb1* (Figure 10) and *Hspa1* (Figure 11) gene promoters that are crucial for successfully ongoing spermatogenesis and their activity is regulated by CpG island methylation in their promoters. Hypomethylation of CpG sites was indicated for all of the investigated promoters, as the level of methylation was between 0–15%. For the *Crem* promoter, there were significant differences detected between groups in DNA methylation for the CpG in position 2 (1.14±0.38 vs. 3.57±1.72) (*p≤0.05) (Figure 9).

Regarding the *Creb1* promoter, Toxo^−^ group showed an elevated methylation pattern compared to the Toxo^+^ group. The other CpGs did not show any statistically significant difference between infected and control animals (p≥0.05). However, we can follow a similar trend of a slightly elevated methylation pattern for all CpGs in the Toxo^−^ group compared to the Toxo^+^ group except for CpG position 4 (Figure 10).

For the *Hspa1* promoter methylation, there were significant differences detected between the Toxo^+^ and Toxo^−^ group in DNA methylation for the CpG in position 3 (7.29±1.60 vs. 5.43±2.94) (*p≤0.05) (Figure 9). For this promoter, there was higher DNA methylation detected in Toxo^+^ compared to the Toxo^−^ group. The other CpGs did not show any statistically significant difference between the infected and control animals (p≥0.05 (Figure 11).

## Discussion

In animals and man, *Toxoplasma* has been found in nearly all organs and symptoms are the same or similar in both animals and humans [23]. Consistent with results obtained on reproductive parameters in mice and rats during the acute stage of the infection [24], [25], humans can also undergo changes in the reproductive system [26]. On the other hand, mechanisms of the changes in reproductive parameters can be also related to the general host-pathogen reactive processes (such as high temperature and subsequent weight loss and/or system inflammation). The clinical manifestation with weight loss, hypothermia, hypoglycemia, hyperkalemia, and increased liver-derived enzymes as well as the onset of cellular and antibody immune response seems to be a common phenomenon for acute parasite infection. Body weight reduction was observed in *Toxoplasma* infected mice during our experiments as well as in several other studies [27], [28]. It is more likely that body weight loss coincided with the host’s immune response, cytokine release and toxic shock-like syndrome, possibly mediated by the systemic TNF-α overproduction [29], [30]. Linkenhoker and Lindton [28] assessed the effects of the nesting material on the body weights of mice infected with *Toxoplasma* and observed that the nesting material did not significantly alter the weights of mice after *Toxoplasma* inoculation, but did decrease the rates of growth prior to inoculation. The body weight decrease may not be exclusively specific to the *T. gondii*, but also to other parasitic protozoe for example *Trypanosoma cruzi* [31], *Leishmania sp,* [32] and *Plasmodium sp*. [33]. However *T. gondii* is so widely spread over the human population that knowledge about its impact on reproduction parameters is of high importance. In particular, in context of idiopathic male subfertility, parasite infection should be taken into consideration as one of the possible reasons of reduced male fertility.

In the *Toxoplasma* positive (Toxo^+^) group compared to the uninfected (Toxo^−^) controls, there were statistically significant differences detected in the reproductive parameters obtained from detailed testicular histometric analysis, a decreased level of LH in urine, and changes in DNA methylation pattern of testicular epigenome.

The level of LH in urine was tested to address the first hypothesis, which suggests that *T. gondii* may activate the hypothalamo-pituitary-adrenal (HPA) stress axis and consequently modify the hypothalamo-pituitary-gonadal (HPG) axis resulting in an altered release of gonadotropins [7], [9]. The fact that according to our results the LH level was significantly decreased in the Toxo^+^ group brings an important indication for understanding the mechanism of how *T. gondii* may interfere with its host. It was observed that male mice infected with the parasite do not increase their sexual attractiveness to females [34], unlike male rats [35]. Results of our experiments support the data suggesting that it is likely that acute stress of infection in mice suppresses gonadal function [34]. Any non-physiological decrease of the LH level may seriously disturb the whole spermatogenesis process, as the LH is one of the main regulators for germ cell development. Due to the fact that LH released from hypophysis is regulated by the hypothalamic GnRH [36], the modification of HPA axis by the infection can result in an altered gonadotropin level due to the decrease of GnRH secretion by the hypothalamus or by the decrease of sensitivity of the hypophysis towards the GnRH [37]. Therefore, the detected decreased LH level in the Toxo^+^ group positively correlates with the results from the testicular morphometric analysis, showing serious alterations in the spermatogenesis process.

The crucial spermatogenesis parameters, such as the number of Sertoli cells per 20 seminiferous tubules, the number of leptotene primary spermatocytes per 250 Sertoli cells (index250), the number of spherical spermatids within 20 seminiferous tubular cross section, and the minor diameter of 50 seminiferous tubules were all modified and gave an overall picture of the status of spermatogenesis in mice under the stress caused by the infection. A statistically significant increase was identified in the number of Sertoli cells per seminiferous tubules in the Toxo^+^ group. It is known that the proliferation of Sertoli cells is determined only to the prenatal and prepubertal stage of mammalian development [38]. Since puberty, the population of Sertoli cells is stable and unchangeable. However, a recent study brings evidence that even the mature population of Sertoli cells can be hormonally modified and the number of cells can be increased [39], [40]. The gonadotropins are involved in the regulation of Sertoli cell proliferation [41] and they are also involved in the timing of the termination of proliferation as it has been shown that mice lacking circulating gonadotropins results in a slow down and time prolonged Sertoli cell proliferation [42]. *In vitro* studies in mice and humans [43] and *in vivo* in hamsters [44] showed that Sertoli cells retain the ability to proliferate and they can actively respond to a decreasing level of gonadotropins by prolonged maturation. This could provide the breach in the blood-testis barrier that would be necessary for *T. gondii* to affect the leptotene spermatocytes and round spermatids. As in natural oral infections, *Toxoplasma* traverses biological barriers such as the blood–testis barrier to reach immunologically privileged sites where it causes the most severe pathology [45].

The number of Sertoli cells reflects the mean diameter of the seminiferous tubules due to the fact that Sertoli cells are the key-constructing components of the seminiferous tubule tissue, as shown previously [46], [47]. In our study, the mean tubule diameter was also significantly increased in the Toxo^+^ group compared to Toxo^−^ group and, moreover, it was positively correlated with the elevated number of Sertoli cells.

On the other hand, the statistically significant decrease of leptotene primary spermatocytes and spherical spermatid was detected and the number of spermatocytes over 250 Sertoli cells (index250) was significantly lowered in the Toxo^+^ group. This drop in the germ cell number is possibly caused by increased apoptosis that is triggered by a decreased gonadotropin level. Elevated apoptosis of male germ cells in *Toxoplasma* infected mice was previously reported [8]. Already Marathe et al. [48] has shown that specific immunoneutralization of LH causes apoptotic cell death of meiotic and post-meiotic germ cells in rat testis. At the same time, a decreased level of gonadotropins and testosterone leads to a decreased proliferation during the spermatogenesis process and spermatocytes are believed to be the most vulnerable stage to be affected [49]. In the mammalian testes, pituitary gonadotropins and testosterone have been shown to regulate germ cell survival, and exposure to deprivation of hormones can lead to cellular apoptosis in the mammalian testis which correlates with our finding of a decreased level of LH as well as lower numbers of spermatocytes and spermatids in Toxo^+^ mice [50].

The second hypothesis ‘How *Toxoplasma gondii* could influence host reproductive parameters’ was based on epigenetic reprogramming of male germ cells and/or somatic testicular cells. There is a newly expanding field of patho-epigenics and it has been shown that several pathogens such as viruses, bacteria, micro and macro parasites, fungi and others trigger specific host cell epigenetic alterations [14].

Up to present, it has not been addressed whether a parasite such as *T. gondii* can also induce pathological changes through specific epigenetic modification, such as aberrant DNA methylation of specific genes, which are crucial for the process of spermatogenesis [51]. Our pilot results from the global testicular methylation, and specific DNA methylation of the selected spermatogenesis crucial genes, has brought about the first evidence that *T. gondii* can modify the host testicular epigenome. Connections between an abnormal DNA methylation pattern and compromised spermatogenesis in humans diagnosed with idiopathic infertility was previously reported [12], [52].

Presented results from testicular global DNA methylation quantification clearly revealed a significantly elevated DNA methylation in the Toxo^+^ group of mice and gave the first indication that *T. gondii* is able to modify its host epigenome. The mechanism in which *T. gondii* manipulates the host epigenetic machinery is still unknown. Brunet et al., [53] demonstrated that UHRF1 (Ubiquitin-like with PHD and ring finger domains 1) plays a central role in the transmission of methylation from mother cells to daughter cells and as a consequence induces host cell cycle arrest at G2, promoting its proliferation. UHRF1 recruits DNA (cytosine-5-)-methyltransferase 1 (DNMT1) to the specific site of the host cell and methylates both DNA strands [54]. In addition, UHRF1 affects epigenetic modifications, including DNA methylation, histone methylation, and chromatin remodelling. Therefore, *T. gondii* may exploit UHRF1 to control the host cell epigenetic machinery [55], [56]. Due to the fact that UHRF1 recruits DNA methylase and also a histone methylase, an aberrant overexpression of UHRF1 may cause DNA hypermethylation of male germ cells. Furthermore, a detailed study is required to classify the selected specific targeted-genes, which are epigenetically regulated. We can report on the results of the following three genes: Hspa1, Crem and Creb1 DNA methylation representing an epigenetic modification of cytosine residues within CpG islands, which is associated with modulation of specific gene expression, was tested. Decreased sperm motility in *Toxoplasma* infected individuals was reported [6], [7]. The epigenetically regulated Hspa1 gene, which codes a testis specific heat shock cognate protein HSC70t expression, is required by postmeiotic germ cells for the assembly and function of protein complexes involved in energy production [19]. Therefore, the reported elevated Hspa1 gene methylation may possibly result in a lowered protein expression contributing to compromised sperm motility reported in *Toxoplasma* affected humans [6] and rats [7].

The CREM and CREB proteins are cyclically expressed at high levels during spermatogenesis in germinal and somatic Sertoli cells and correlate with the fluctuations in cAMP signaling, which is induced by the pituitary-gonadotropin hormones LH and FSH both during sexual maturation of testis and during the 12-day cycles of spermatogenesis that occur in adult testis [17]. It is suspected that the reported decreased LH level in Toxo^+^ mice could possibly trigger misbalanced CREM and CREB expression via modification of cAMP signaling. However, the epigenetically modified Crem or Creb1 gene expression can also lead to abnormal protein transcription, resulting in modified signaling pathways, including gonadotropins.

A reduction of spermatogenesis and a decrease of sperm motility and quality was reported in correlation with abnormal Crem methylation [18]. Moreover, it was shown that CREB is required to produce a Sertoli cell-derived factor that is critical for germ cell survival [57] and increased apoptosis of meiotic and postmeiotic male germ cells is increased in the case of Creb314-deficient mice [20].

CREM activators are expressed in post-meiotic haploid germ cells and are essential for spermatid maturation [58]. We are reporting on the statistically significant decrease of Crem DNA methylation in one CpG position in the Toxo^+^ group compared to the Toxo^−^ group. However, it remains to be clarified, whether the detected decreased methylation in Crem CpG in the specific position would lead to an abnormal increased expression of CREM protein and consequently to a disturbance of the spermatogenesis and spermatid maturation observed in immunohistochemical analysis of the Toxo^+^ mouse testis.

CREB proteins have an ability to respond to various stress conditions and maintain cellular homeostasis [59], [60]. For this reason, their dynamic action during *Toxoplasma* infection may be suspected. In Sertoli cells, the level of CREB protein fluctuates in cyclic waves, and it is dependent on specific cell association during spermatogenesis [16]. CREB activity is regulated by phosphorylation in seminiferous tubules of the adult testis according to the spermatogenic cycle and can rapidly react to the current state of the organism [61].

Our data from *Creb1* quantitative DNA methylation by pyrosequencing show a very low level of CpGs methylation (0–5%) and there was no significant difference in methylation between the Toxo^+^ and Toxo^−^ group. In contrast to global methylation analysis, pyrosequecing data provide more accurate results on the methylation level with resolution to each single cytosine, however, the sequenced PCR product covers less than 80 bp. Thus, genome wide methylation analysis, for example shotgun sequencing, would be more appropriate in order to identify the spermatogenesis specific gene candidates, which could show *Toxoplasma* related changes of methylation status. Further study on mRNA and protein level would be also required to clarify the mechanism of epigenetic action for the quality of spermatogenesis.

The goal of this study was to show a novel approach and results connecting *T. gondii* infection with compromised male reproductive fitness. The final outcome of this experimental layout is, that toxoplasma-infected males produced diminished numbers of sperm, but they were not sterile. A further investigations will be needed in order to elaborate breeding of toxo positive males with control female, toxo positive females with control males. Moreover, a large-scale experiment with greater number of animals and generations will be needed to observe the statistically significant effect.

The communication between somatic and male germinal cells is crucial for their proliferation, differentiation and maturation and its regulation happens on several levels, including hormonal and epigenetic. Any disturbance of this fragile system can be reflected in the abnormal phenotype, such as of testis morphology or fertility status for example. Whether, reported changes in *Toxoplasma* infected mice are permanent or reversible, as well as whether, they reflect a universal mechanism for *Toxoplasma gondii* modification of its mammalian hosts, remains to be identified.

## Supporting Information

Figure S1 Cell type dependence graph. The 3D distance weighted graph shows a dependence of spermatid abundance (z axis) in the tubules on the combined abundance of leptotene primary spermatocytes (x axis) and Sertoli cells (y axis). The stiffness 0.1 was set, the different colors in the legend indicate the different number of spermatid in the appropriate area. The red broken circle indicates the major difference between the Toxo^+^ and Toxo^−^ groups.
(TIF)

Table S1 Intraclass correlation.
(DOCX)
