# Supplementary figures and images for: Toxoplasma gondii Decreases the Reproductive Fitness in Mice
Source: PLoS One. 2014 Jun 18;9(6):e96770. doi: 10.1371/journal.pone.0096770 (PMC4062421; doi:10.1371/journal.pone.0096770)

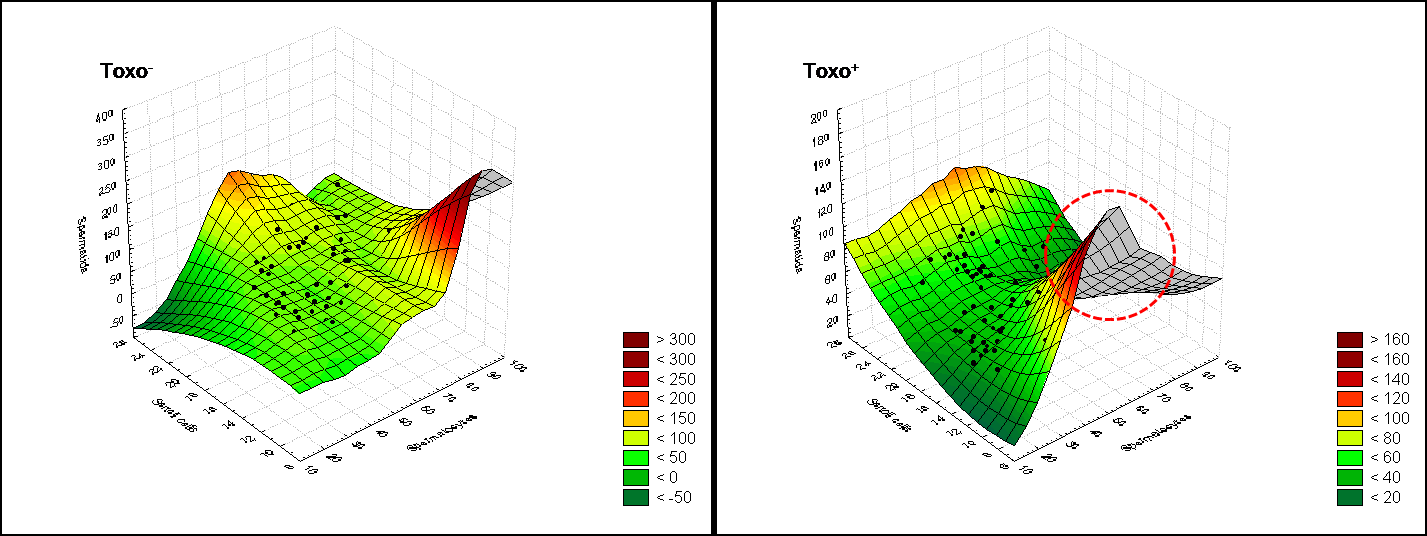

Supplement: Figure S1 — Cell type dependence graph. The 3D distance weighted graph shows a dependence of spermatid abundance (z axis) in the tubules on the combined abundance of leptotene primary spermatocytes (x axis) and Sertoli cells (y axis). The stiffness 0.1 was set, the different colors in the legend indicate the different number of spermatid in the appropriate area. The red broken circle indicates the major difference between the Toxo+ and Toxo− groups. (TIF) [file pone.0096770.s001.tif]
