# Supplementary material for: Toxoplasma gondii Decreases the Reproductive Fitness in Mice
Source: PLoS One. 2014 Jun 18;9(6):e96770. doi: 10.1371/journal.pone.0096770 (PMC4062421; doi:10.1371/journal.pone.0096770)
Supplement: Table S1 — Intraclass correlation. (DOCX) [file pone.0096770.s002.docx]

Suplementary files

| **Table S1**. Intraclass correlation. | | | | | | | | |
| --- | --- | --- | --- | --- | --- | --- | --- | --- |
| **Group** | **Parameter** | **Mean** | **SD** | **Min** | **Max** | **N** | **ICC** | **(95% C.I.)** |
|  | SC (No.) | 17.6 | 4.0 | 8.0 | 26.0 | 160 | .780 | (.479, .948) |
|  | LS (No.) | 35.5 | 12.8 | 20.0 | 89.0 | 160 | .764 | (.440, .944) |
| Toxo^+^ | S (No.) | 47.4 | 20.3 | 14.0 | 101.0 | 160 | .866 | (.683, .968) |
|  | TD (µm) | 131.5 | 32.2 | 58.2 | 223.1 | 400 | .836 | (.620, .961) |
|  | Index250 | 506.9 | 151.4 | 192.3 | 1285.7 | 160 | .763 | (.438, .943) |
|  | SC (No.) | 15.2 | 3.2 | 9.0 | 24.0 | 160 | .807 | (.541, .954) |
|  | LS (No.) | 43.6 | 15.0 | 20.0 | 89.0 | 160 | .934 | (.844, .984) |
| Toxo^-^ | S (No.) | 66.8 | 23.9 | 29.0 | 119.0 | 160 | .684 | (.251, .925) |
|  | TD (µm) | 121.4 | 29.9 | 63.3 | 241.7 | 400 | .894 | (.754, .974) |
|  | Index250 | 722.6 | 198.2 | 343.6 | 1350.0 | 160 | .807 | (.542, .954) |
